# Propranolol-induced inhibition of unconditioned stimulus-reactivated fear memory prevents the return of fear in humans

**DOI:** 10.1038/s41398-020-01023-w

**Published:** 2020-10-13

**Authors:** Jiahui Deng, Le Shi, Kai Yuan, Ping Yao, Sijing Chen, Jianyu Que, Yimiao Gong, Yanping Bao, Jie Shi, Ying Han, Hongqiang Sun, Lin Lu

**Affiliations:** 1 Peking University Sixth Hospital, Peking University Institute of Mental Health, NHC Key Laboratory of Mental Health (Peking University), National Clinical Research Center for Mental Disorders (Peking University Sixth Hospital), Chinese Academy of Medical Sciences Research Unit (No.2018RU006), Peking University, Beijing, China; 2 Basic Medical College, Inner Mongolia Medical University, Hohhot, China; 3 Faculty of Medicine, Department of Psychiatry, Chinese University of Hong Kong, Sha Tin, Hong Kong SAR, China; 4 National Institute on Drug Dependence and Beijing Key Laboratory on Drug Dependence Research, Peking University, Beijing, China; 5 Peking-Tsinghua Center for Life Sciences and PKU-IDG/McGovern Institute for Brain Research, Peking University, Beijing, China

**Keywords:** Human behaviour, Psychiatric disorders

## Abstract

Fear memories can be reactivated by a fear-associated conditioned stimulus (CS) or unconditioned stimulus (US) and then undergo reconsolidation. Propranolol administration during CS retrieval-induced reconsolidation can impair fear memory that is specific to the reactivated CS. However, from a practical perspective, the US is often associated with multiple CSs, and each CS can induce a fear response. The present study sought to develop and test a US-based memory retrieval interference procedure with propranolol to disrupt the original fear memory and eliminate all CS-associated fear responses in humans. We recruited 127 young healthy volunteers and conducted three experiments. All of the subjects acquired fear conditioning, after which they received the β-adrenergic receptor antagonist propranolol (40 mg) or placebo (vitamin C) and were exposed to the US or CS to reactivate the original fear memory. Fear responses were measured. Oral propranolol administration 1 h before US retrieval significantly decreased subsequent fear responses and disrupted associations between all CSs and the US. However, propranolol administration before CS retrieval only inhibited the fear memory that was related to the reactivated CS. Moreover, the propranolol-induced inhibition of fear memory reconsolidation that was retrieved by the US had a relatively long-lasting effect (at least 2 weeks) and was also effective for remote fear memory. These findings indicate that the US-based memory retrieval interference procedure with propranolol can permanently decrease the fear response and prevent the return of fear for all CSs in humans. This procedure may open new avenues for treating fear-related disorders.

## Introduction

Strong emotional stimuli, such as traumatic experiences in our daily lives, may induce pathological changes that usurp the normal nervous systems of learning and memory, resulting in the formation of excessive and long-lasting maladaptive emotional memories that underlie anxiety and fear-related disorders^1–3^. In the laboratory, Pavlovian fear conditioning models are used to explore neurobiological mechanisms and new translational treatments for anxiety and fear-related disorders^4,5^. Pavlovian fear conditioning is a behavioral paradigm in which an initially neutral conditioned stimulus (CS), usually a tone or picture, is paired with a noxious unconditioned stimulus (US) that elicits an unconditioned fear response. Fear memory reconsolidation is the process by which reactivation by exposure to the CS or US makes memory traces labile, thereby triggering transient protein destabilization that can be modified by pharmacological and behavioral interventions for several hours after memory reactivation^6–8^. The reconsolidation process enables fear memories to be updated with new information.

Pharmacological manipulation of memory reconsolidation has been well documented^7,9^ and opens up a promising new avenue for treating anxiety disorders. However, most of the agents used during reconsolidation interference in animals are not feasible for human studies, except for some well-tolerated, nontoxic drugs, such as the β-adrenergic receptor antagonist propranolol. Furthermore, previous studies found that reconsolidation requires activation of the noradrenergic system^10,11^. Administration of the β-adrenergic receptor (β-AR) antagonist propranolol combined with memory retrieval significantly decreased physiological responses to fear-related cues in healthy volunteers^12–14^. Nevertheless, such a beneficial effect was not observed in other studies^15^. One reason for this inconsistency might be related to the different inclusion and exclusion criteria or different modes of memory activation and interventions. Therefore, the efficacy of propranolol-induced interference after retrieval in decreasing fear memory needs further validation.

One potential reason for the limited efficacy of propranolol on fear memory is that previous studies exposed subjects to fear-related CSs and not the US to retrieve fear memory into reconsolidation. Exposure to the US can also trigger memory reconsolidation and may have a different neural basis compared with CS retrieval^16^. Our previous studies found that extinction training after CS or US retrieval inhibited the response to the reactivated CS or all CSs^17,18^. We also developed a US-based memory retrieval procedure with propranolol in smokers and found that oral propranolol administration before nicotine US-triggered memory retrieval decreased subsequent nicotine preference that was induced by all nicotine CSs and nicotine craving^19^. Additionally, animal studies found that the disruption of protein synthesis or β-AR blockade after US retrieval decreased the expression of fear that was induced by multiple CSs^16,20^. These studies suggest that US retrieval may activate multiple memory traces, and behavioral and drug interventions during this period may be more effective than CS retrieval. In the present study, we tested whether US-based memory retrieval interference with propranolol decreases the fear response to multiple CSs and prevents the return of fear in humans.

## Subjects and methods

### Participants

One hundred twenty-seven volunteers were enrolled in the study through posters and advertisements. The inclusion criteria were the following: (1) between 18 and 35 years of age and (2) generally good health as determined by a physician. The exclusion criteria were the following: (1) current or past history of medical or psychiatric illness, diagnosed by the Structured Clinical Interview for *Diagnostic and Statistical Manual of Mental Disorders*, 4th edition, Axis I Disorders (SCID), (2) the use of medications, (3) contraindications to the use of propranolol, such as bronchial asthma, cardiac shock, heart block, severe heart failure, sinus bradycardia, and blood pressure <90/60 mmHg, and (4) had participated or were participating in other electric shock-related fear memory experiments. All of the participants were scheduled for a screening interview, during which they were told further details about the experimental protocol and signed an informed consent form. The study was approved by the Institutional Review Board of Peking University Sixth Hospital. Each participant was paid USD$50 for their participation. During the baseline session before the experiments, all of the participants completed questionnaires to collect basic demographic information, including sex, age, education, height, weight, and body mass index (BMI). To control for the possible confounding effect of cognitive impairment, we assessed baseline cognitive function using the Montreal Cognitive Assessment (MoCA) and digit span test^21^.

### Fear conditioning

The protocol was based on our previous studies^17,22^. Before fear conditioning, the subjects determined the US intensity themselves, beginning at a very mild level of shock (20 V), the intensity of which gradually increased until the shock reached the maximum level that the subjects felt uncomfortable but not painful (the highest level was 100 V). All of the shocks were given for 200 ms, with a current of 50 pulses per second.

For fear conditioning, the participants were instructed to pay attention to the computer screen and try to determine the relationship between the different CSs (colored square pictures) and the US (a mild electric shock to the wrist). The CSs were presented for 4 s with a variable interval of 8–12 s.

In Experiment 1, two-colored squares (CS^+^ and CS^–^) were used. The CSs^+^ were paired with the US under a partial reinforcement schedule (50% reinforced). The CS^–^ was never paired with the US. Fear acquisition consisted of eight non-reinforced presentations of each CS, intermixed with an additional eight CS^+^ presentations that co-terminated with an electric shock. Three presentations were included in one trial. During each trial, the order of presentation of the reinforced CS^+^, non-reinforced CS^+^, and CS^–^ was randomized. To counteract the effect of color on an individual’s memory, we used red squares as the CS^+^ and yellow squares as the CS^–^ for 31 participants (No retrieval + propranolol [*n* = 7], US retrieval + propranolol [*n* = 8], US retrieval + placebo [*n* = 8], and US retrieval + 8 h + propranolol [*n* = 8]), while used yellow squares as the CS^+^ and red squares as the CS^–^ for 29 participants (No retrieval + propranolol [*n* = 7], US retrieval + propranolol [*n* = 7], US retrieval + placebo [*n* = 8], and US retrieval + 8 h + propranolol [*n* = 7]). The application of counterbalancing combination of participants was based on their odd/even order of inclusion.

In Experiments 2 and 3, three colored squares (CS1, CS2, and CS^–^) were used. Two squares (CS1 and CS2) were paired with the US under a 50% reinforcement schedule. Acquisition consisted of eight reinforced presentations of CS1 and CS2, eight non-reinforced presentations of CS1 and CS2, and eight presentations of the CS^–^. Five presentations were included in one trial. During each trial, the order of presentation of reinforced CS1, non-reinforced CS1, reinforced CS2, non-reinforced CS2, and CS^–^ was randomized. Similar to Experiment 1, to counteract the effect of color on an individual’s memory, the colors (red, blue, or yellow) of squares as the CS1, CS2, and CS^–^ were randomized among the participants.

### Retrieval and intervention

The dose of propranolol (40 mg, p.o.; YABANG Pharma) was chosen based on previous studies^19,23^. A single dose of 40 mg propranolol or placebo (vitamin C) was administered 1 h before the memory retrieval manipulation or 8 h after retrieval. The 1 h interval between propranolol administration and retrieval was based on previous studies^13,24^ and coincided with the pharmacodynamics of propranolol^25^. The 8 h interval between drug intake and retrieval was outside the reconsolidation window^26^. During US reactivation, a weaker electric shock was administered, the intensity of which was half the intensity that was used in fear conditioning^17^. During CS reactivation, the non-reinforced CS^+^ was presented once.

In Experiment 1, 1 day after acquisition, fear memory was reactivated by exposure to the CS^+^ or US. The subjects in the US retrieval + propranolol group were administered propranolol 1 h before reactivation by a weaker electric shock (to confirm that propranolol exerted its actions within the reconsolidation time window). One group only received propranolol 1 day after fear memory acquisition without exposure to the CS^+^ or US (No retrieval + propranolol group). The US retrieval + placebo group was administered placebo 1 h before a weaker electric shock was applied. Subjects in the US retrieval + 8 h + propranolol group were given propranolol 8 h after US reactivation (to confirm that propranolol exerted its actions outside the reconsolidation time window). The fear memory test and reinstatement test occurred 24 h after the intervention.

In Experiment 2, 1 day after acquisition, fear memory was reactivated by exposure to either CS1 or the US. In the CS1 retrieval + propranolol group, propranolol was administered 1 h before the fear response that was elicited by CS1. Participants in the US retrieval + propranolol group received propranolol, and the US retrieval was applied 1 h later. The fear memory test and reinstatement test were performed both 24 h and 2 weeks after the intervention.

In Experiment 3, 2 weeks after acquisition, fear memory was reactivated by exposure to the US (a weaker electric shock). Placebo or propranolol was administered 1 h before US retrieval. The test procedures were the same as in Experiment 2.

### Test

Two tests were performed in which the participants were presented with non-reinforced presentations of the CS. One minute after the fear memory test, the participants received three unsignaled US presentations, followed by the reinstatement test. During the tests, the CSs were presented for 4 s, followed by an inter-stimulus interval of 8–12 s, during which the participants looked at a fixation point on the computer screen. In Experiment 1, 10 CS^+^ and 10 CS^–^ were presented in the fear memory test, and 8 CS^+^ and 8 CS^–^ were presented in the reinstatement test. In Experiments 2 and 3, 10 CS1, 10 CS2, and 10 CS^–^ were presented during the fear memory test, and 8 CS1, 8 CS2, and 8 CS^–^ were presented during the reinstatement test.

In Experiments 1 and 3, the fear memory test and reinstatement test were conducted 24 h after the intervention. In Experiment 2, the fear memory test and reinstatement test were conducted both 24 h and 2 weeks after the intervention.

### Experimental design

In the present study, we used a single-blind experimental design, in which the experimenters were aware of the administration of propranolol or placebo, but the participants were not. The specific experimental designs are described below.

Experiment 1 included 60 participants. They were randomly assigned to one of four groups using random numbers: No retrieval + propranolol (*n* = 14), US retrieval + propranolol (*n* = 15), US retrieval + placebo (*n* = 16), and US retrieval + 8 h + propranolol (*n* = 15). Experiment 1 consisted of fear conditioning, manipulation, a fear memory test, and a reinstatement test.

Experiment 2 included 32 participants. They were randomly assigned to two groups using random numbers: CS1 retrieval + propranolol (*n* = 17) and US retrieval + propranolol (*n* = 15). Experiment 2 consisted of fear conditioning, manipulation, a fear memory test, and a reinstatement test. For the 2-week follow-up experiment, the same participants were recruited to complete a fear memory test and a reinstatement test. Two participants dropped out of the study because of taking curriculum evaluation. Thus 30 participants (CS1 retrieval + propranolol [*n* = 16] and US retrieval + propranolol [*n* = 14]) completed the follow-up.

Experiment 3 included 35 participants. They were randomly assigned to two groups using random numbers: US retrieval + placebo (*n* = 17) and US retrieval + propranolol (*n* = 18). Experiment 3 consisted of fear conditioning, manipulation, a fear memory test, and a reinstatement test.

### Psychophysiological stimulation and assessment

Electric shocks were delivered by a constant-current STM200 stimulator (BIOPAC Systems, Goleta, CA, USA). A stimulating electrode was attached to the right inner wrist. Stimulus presentation was controlled by a computer using E-Prime software (Psychology Software Tools, Sharpsburg, PA, USA). The fear response was assessed by the skin conductance response (SCR), which was recorded through shielded Ag-AgCl electrodes that were attached to the second and third fingers of the left hand. Skin conductance response waveforms were measured using a BIOPAC MP150 system and analyzed using AcqKnowledge 4.0 software (BIOPAC Systems, Goleta, California). These values were then square root-transformed to normalize the distribution.

### Statistical analysis

The results are presented as the mean ± standard error of the mean (SEM) and were analyzed using mixed-model analysis of variance (ANOVA) with appropriate between- and within-subjects factors (see “Results”). We performed post hoc analyses of significant effects in the ANOVAs using the Least Significant Difference test or *t*-test. Values of *p* < 0.05, two-tailed, were considered statistically significant.

For the fear acquisition analysis, reinforced CSs^+^, which induced a strong SCR by an unconditioned shock and confounded the learned fear response, were not included. The last three trials of non-reinforced CSs were used to assess fear acquisition, and fear memory tests were assessed with CSs during the first three trials. To accurately evaluate the return of fear memory, we compared differential SCRs during the first three trials of the reinstatement test with differential SCRs during the last three trials of the fear memory test.

The greatest base-to-peak change in the SCR in a 0–6 s time window after each CS onset was recorded. The differential SCR was assessed by subtracting responses to the CS^–^ from responses to the CS^+^ in corresponding trials. The differential scores were averaged across participants. All of the statistical analyses were performed using SPSS 20.0 software (SPSS, Chicago, IL, USA).

## Results

### Propranolol disrupts the reconsolidation of fear memory after US retrieval

**Fig. 1:**
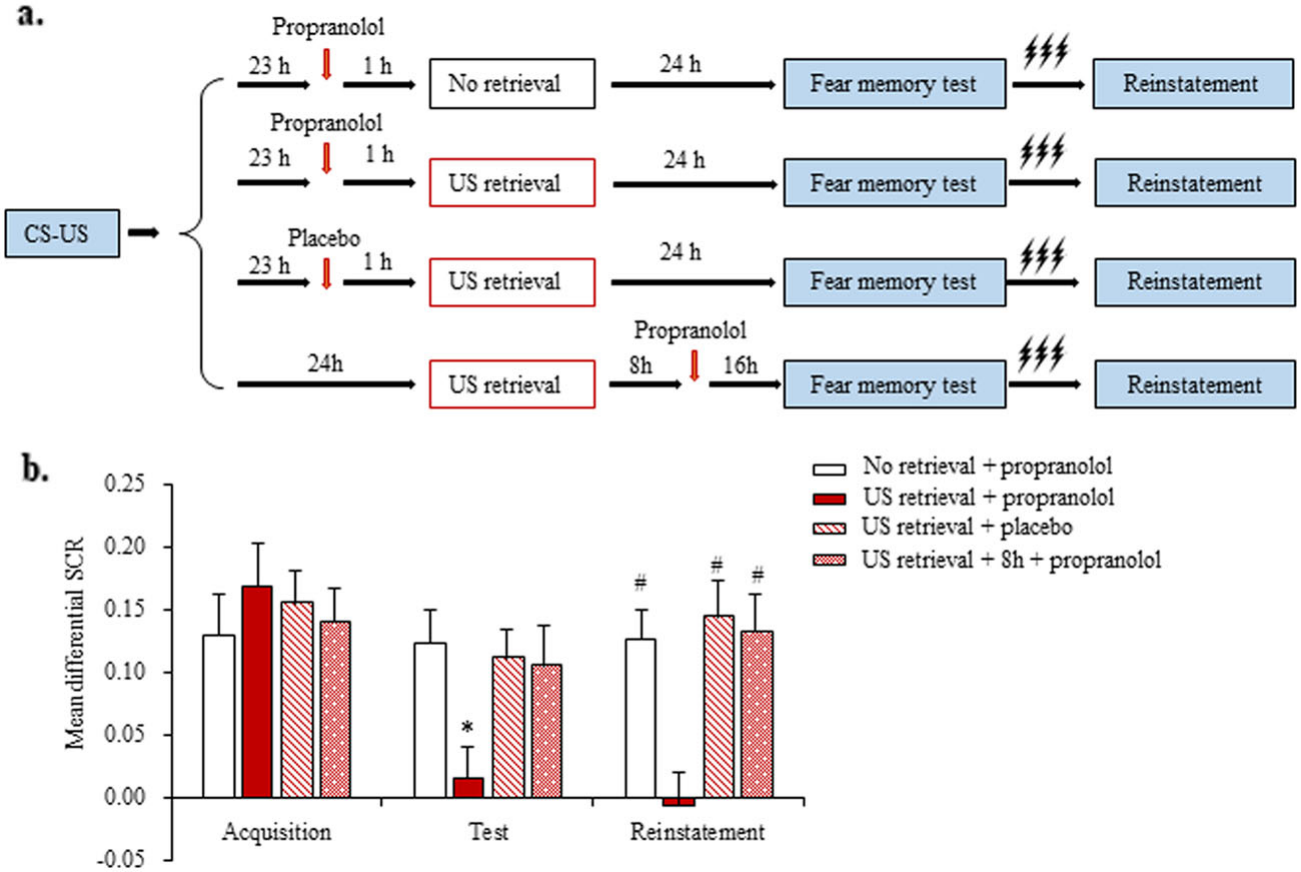
Propranolol administration before unconditioned stimulus (US) retrieval reduced fear expression and fear reinstatement. **a** Experimental design and timeline of Experiment 1. **b** Mean differential skin conductance response (SCR; CS^+^ minus CS^–^) during fear acquisition, the fear memory test, and the reinstatement test in each of the experimental groups (No retrieval + propranolol [*n* = 14], US retrieval + propranolol [*n* = 15], US retrieval + placebo [*n* = 16], and US retrieval + 8 h + propranolol [*n* = 15]). *p < 0.05, comparisons between acquisition and first three trials of test; ^#^*p* < 0.05, comparisons between last three trials of test and reinstatement (all within-group). The data are expressed as mean ± SEM.

In Experiment 1, we first explored whether administration of the β-AR antagonist propranolol before US retrieval disrupts fear memory reconsolidation (Fig. 1a). No significant differences in sex, age, education, height, weight, BMI, MoCA score, digit span test score (forward and backward), or shock intensity were found among the four groups (all *p* > 0.05; Supplementary Table S1). Mean differential SCRs were extracted during fear acquisition (last three non-reinforced trials), the fear memory test (first three trials and last three trials), and the reinstatement test (first three trials). All of the participants in the four groups achieved successful and comparable fear acquisition (mean differential SCR > 0.1; Fig. 1b).

To investigate the effect of oral propranolol administration after US retrieval on fear expression, the participants underwent tests 24 h after fear conditioning. The mixed-model ANOVA, with group (No retrieval + propranolol, US retrieval + propranolol, US retrieval + placebo, and US retrieval + 8 h + propranolol) as the between-subjects factor and time (acquisition and fear memory test [first three trials]) as the within-subjects factor, revealed a main effect of time (*F*_1,56_ = 13.253, *p* = 0.001) and a group × time interaction (*F*_3,56_ = 3.601, *p* = 0.019) but no main effect of group (*F*_3,56_ = 0.641, *p* = 0.592). The post hoc analysis showed that the mean differential SCR decreased in the US retrieval + propranolol group (*p* < 0.05) but not in the other three groups in the fear memory test (all *p* > 0.05; Fig. 1b). The one-way ANOVA showed that the mean differential SCR in the last three trials of the fear memory test was similar in all four groups (*p* > 0.05).

During reinstatement, the mixed-model ANOVA, with group (No retrieval + propranolol, US retrieval + propranolol, US retrieval + placebo, and US retrieval + 8 h + propranolol) as the between-subjects factor and time (fear memory test [last three trials] and reinstatement) as the within-subjects factor, revealed main effects of time (*F*_1,56_ = 32.056, *p* < 0.001) and group (*F*_3,56_ = 4.663, *p* = 0.006) and a group × time interaction (*F*_3,56_ = 3.309, *p* = 0.027). The *post hoc* analysis showed that fear responses occurred in the No retrieval + propranolol, US retrieval + placebo, and US retrieval + 8 h + propranolol groups (all *p* < 0.05; Fig. 1b).

Altogether, these results indicate that propranolol administration within the time window of fear memory reconsolidation after US retrieval decreased fear expression and inhibited the return of fear. Moreover, both propranolol treatment within the time window of reconsolidation and the US reactivation of fear memory appeared to be necessary for the inhibition of fear memory.

### Unconditioned stimulus-based memory retrieval interference procedure with propranolol impairs multiple fear-related memories

**Fig. 2:**
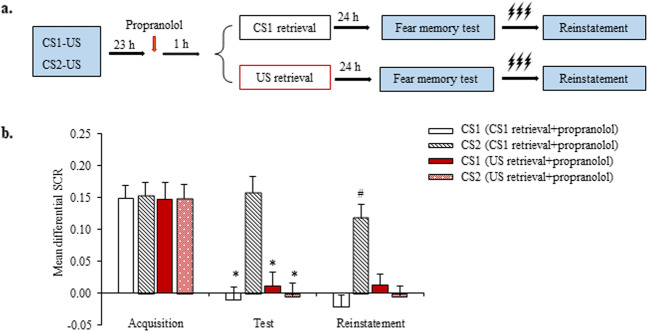
Propranolol-induced disruption of the unconditioned stimulus (US)-triggered reconsolidation of memory for multiple conditioned stimuli (CSs). **a** Experimental design and timeline of Experiment 2. **b** Mean differential skin conductance response (SCR; CS^+^ minus CS^–^) during fear acquisition, the fear memory test, and the reinstatement test for each of the experimental groups (CS1 retrieval + propranolol [*n* = 17], US retrieval + propranolol [*n* = 15]). *p < 0.05, comparisons between acquisition and first three trials of test; ^#^*p* < 0.05, comparisons between last three trials of test and reinstatement (all within-group). The data are expressed as mean ± SEM.

In Experiment 2, we investigated whether oral propranolol administration 1 h before US exposure destabilized multiple CSs that were associated with the US (Fig. 2a). No differences in sex, age, education, height, weight, BMI, MoCA score, digit span test score (forward and backward), or shock intensity were found between the CS1 retrieval + propranolol group and US retrieval + propranolol group (all *p* > 0.05; Supplementary Table S2). During fear acquisition, both groups achieved successful fear responses, with no significant difference between groups (all *p* > 0.05; Fig. 2b).

The mixed-model ANOVA, with group (CS1 retrieval + propranolol and US retrieval + propranolol) as the between-subjects factor and CS (CS1 and CS2) and time (acquisition and fear memory test [first three trials]) as the within-subjects factors, revealed main effects of time (*F*_1,60_ = 60.701 *p* = 0.000), CS (*F*_1,60_ = 5.349, *p* = 0.024), and group (*F*_1,60_ = 4.233, *p* = 0.044) and a significant group × CS × time interaction (*F*_1,60_ = 10.565, *p* = 0.002). Follow-up *t*-tests showed that mean differential SCRs to CS1 and CS2 significantly decreased in the US retrieval + propranolol group (both *p* < 0.05; Fig. 2b). However, significant conditioned fear expression in response to CS2 was detected in the CS1 retrieval + propranolol group (*p* > 0.05; Fig. 2b). The mixed-design ANOVA showed that fear responses to both CSs in the last three trials of the fear memory test were similar between groups (all *p* > 0.05).

Reinstatement was assessed using a mixed-model ANOVA, with group (CS1 retrieval + propranolol and US retrieval + propranolol) as the between-subjects factor and CS (CS1 and CS2) and time (fear memory test [last three trials] and reinstatement) as the within-subjects factors. This analysis showed main effects of group (*F*_1,60_ = 5.125, *p* = 0.027) and CS (*F*_1,60_ = 4.375, *p* = 0.041) and a significant group × CS × time interaction (*F*_1,60_ = 6.411, *p* = 0.014). Follow-up *t*-tests showed the significant reinstatement of conditioned fear in response to CS2 in the CS1 retrieval + propranolol group (*p* < 0.05). No reinstatement was observed in the US retrieval + propranolol group (both *p* > 0.05; Fig. 2b). These results indicate that the disruption of US retrieval-triggered reconsolidation with propranolol inhibited fear responses to multiple CSs.

### Blockade of conditioned fear is maintained for at least 2 weeks

**Fig. 3:**
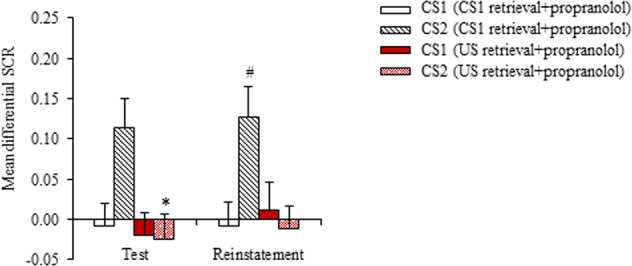
Persistence of the blockade of fear responses to both conditioned stimulus 1 (CS1) and CS2 by the unconditioned stimulus (US)-based memory retrieval interference procedure with propranolol. Mean differential skin conductance response (SCR; CS1^+^ minus CS^–^ or CS2^+^ minus CS^–^) during the fear memory test and reinstatement test after the intervention 2 weeks later. *p < 0.05, comparisons between CS1 retrieval + propranolol (*n* = 16) and US retrieval + propranolol (*n* = 14; between-group); #*p* < 0.05, comparisons between last three trials of test and reinstatement (within-group). The data are expressed as mean ± SEM.

Two weeks later, 30 participants from Experiment 2 were invited to return to the laboratory for tests to assess the long-term effect of the US-based memory retrieval interference procedure with propranolol. Higher conditioned fear expression in response to CS2 was detected in the CS1 retrieval + propranolol group compared with the US retrieval + propranolol group in the fear memory test (*t*_28_ = 2.288, *p* = 0.030; Fig. 3). The mixed-model ANOVA, with group (CS1 retrieval + propranolol and US retrieval + propranolol) as the between-subjects factor and CS (CS1 and CS2) and time (fear memory test [last three trials] and reinstatement) as the within-subjects factors, revealed significant main effects of group (*F*_1,56_ = 6.590, *p* = 0.013) and CS (*F*_1,56_ = 4.110, *p* = 0.047) and a group × CS × time interaction (*F*_1,56_ = 5.999, *p* = 0.017). Follow-up *t*-tests showed significantly higher conditioned fear in response to CS2 in the CS1 retrieval + propranolol group in the reinstatement test (*p* < 0.05). No reinstatement was observed in the US retrieval + propranolol group (both *p* > 0.05; Fig. 3). These results indicate that the disruption of fear reconsolidation after US retrieval with propranolol led to long-lasting blockade of the return of fear in response to all CSs.

### Propranolol-induced disruption of US-triggered reconsolidation inhibits remote fear memory

**Fig. 4:**
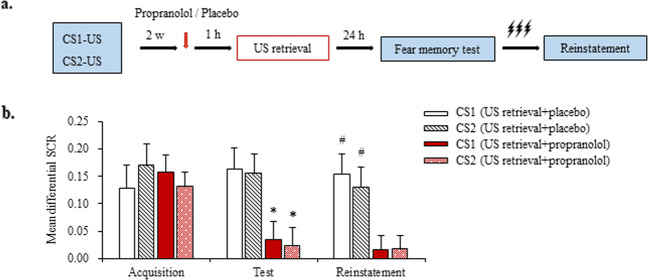
Propranolol disrupts the unconditioned stimulus (US)-triggered reconsolidation of remote fear memory. **a** Experimental design and timeline of Experiment 3. **b** Mean differential skin conductance response (SCR; CS^+^ minus CS^–^) during fear acquisition, the fear memory test, and the reinstatement test for each of the experimental groups (US retrieval + placebo [*n* = 17], US retrieval + propranolol [*n* = 18]). *p < 0.05, comparisons between acquisition and first three trials of test; #*p* < 0.05, comparisons between last three trials of test and reinstatement (all within-group). The data are expressed as mean ± SEM.

In Experiment 3, we investigated whether propranolol administration during US-triggered reconsolidation disrupts remote fear memory (Fig. 4a). The participants first underwent fear conditioning and were treated with propranolol 1 h before US retrieval 2 weeks later. Tests were conducted 24 h after the manipulation. No differences in sex, age, education, height, weight, BMI, MoCA score, digit span test score (forward and backward), or shock intensity were found between the US retrieval + placebo group and US retrieval + propranolol group (all *p* > 0.05; Supplementary Table S3). During fear acquisition, all of the participants in the two groups achieved successful and comparable acquisition (mean differential SCR > 0.1; Fig. 4b).

The mixed-model ANOVA, with group (US retrieval + placebo and US retrieval + propranolol) as the between-subjects factor and CS (CS1 and CS2) and time (acquisition and fear memory test [first three trials]) as the within-subjects factors, revealed main effects of group (*F*_1,66_ = 4.877, *p* = 0.031) and time (*F*_1,66_ = 6.470, *p* = 0.013) and a group × time interaction (*F*_1,66_ = 5.544, *p* = 0.022) but no group × CS × time interaction (*F*_1,66_ = 0.383, *p* = 0.538). In the fear memory test, fear responses to CS1 and CS2 significantly decreased in the US retrieval + propranolol group (both *p* < 0.05) but not in the US retrieval + placebo group (both *p* > 0.05; Fig. 4b). The mixed-design ANOVA showed that the responses to CS1 and CS2 in both groups were similar in the last three trials of the fear memory test (all *p* > 0.05).

The mixed-model ANOVA, with group (US retrieval + placebo and US retrieval + propranolol) as the between-subjects factor and CS (CS1 and CS2) and time (fear memory test [last three trials] and reinstatement) as the within-subjects factors, revealed main effects of group (*F*_1,66_ = 6.849, *p* = 0.011) and time (*F*_1,66_ = 4.839, *p* = 0.031) and a group × time interaction (*F*_1,66_ = 8.906, *p* = 0.004) but no group × CS × time interaction (*F*_1,66_ = 0.02, *p* = 0.888). The significant reinstatement of conditioned fear in response to CS1 and CS2 was observed in the US retrieval + placebo group (both *p* < 0.05). No reinstatement was observed in the US retrieval + propranolol group (both *p* > 0.05; Fig. 4b). Altogether, these results suggest that the US-based memory retrieval interference procedure with propranolol also effectively disrupted remote fear memory.

## Discussion

**Fig. 5:**
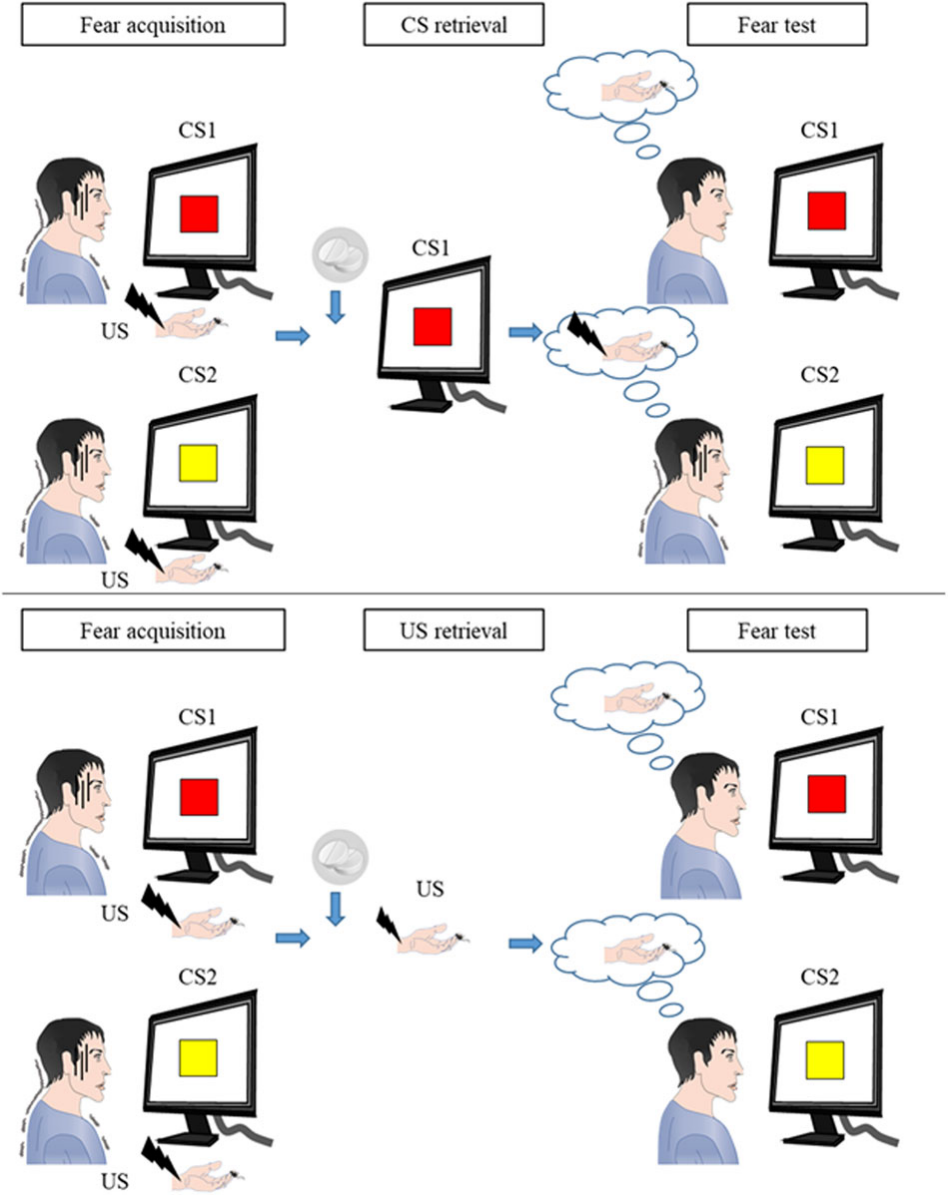
Schematic model of propranolol-induced disruption of reactivated fear memories. Propranolol administration before CS1 retrieval only disrupts the CS1-US association (e.g., the association between the red square and shock), leaving the associations between other CSs and the US (e.g., the association between the yellow square and shock) intact. In contrast, propranolol administration before US retrieval disrupts the associations between all CSs and the US.

The present study found that disrupting US-induced memory reconsolidation with the β-AR blocker propranolol resulted in the inhibition of fear memory. Conditioned stimulus-based memory retrieval interference targets only one kind of CS. In contrast, US-based memory retrieval interference with propranolol disrupted associations between all of the CSs and the US (Fig. 5). Moreover, this manipulation with propranolol led to a long-lasting blockade of the return of fear. This paradigm inhibited both recent fear memory and remote fear memory. The findings indicate that the US-based memory retrieval interference procedure with propranolol may have translational potential for the treatment of anxiety and fear-related disorders (e.g. spider phobia, acrophobia, and social anxiety).

In the present study, propranolol appeared to work during the reconsolidation time window, which lasted ~6 h, during which the original memory could be updated with new protein synthesis^26–28^. However, the fear memory was impervious to the amnestic effects of propranolol when it was administered 8 h after memory reactivation, during which memory enters a steady state. These results were consistent with our previous study that found that β-AR blockade during the temporal window of reconsolidation inhibited memory, with no effect beyond this time window^19^. One key issue with the pharmacological manipulation of reconsolidation is the timing of propranolol administration. Previous studies showed that propranolol administration 1 h before reactivation but not 2 h after reactivation decreased fear responses to the reactivated CS^14^. Propranolol administration immediately after learning or retrieval did not disrupt the consolidation or reconsolidation of fear memory in humans^29^. Furthermore, a previous study found that propranolol administration 1 h before memory reactivation decreased the fear response but did not affect memory retrieval^30^. Thus, we chose to administer propranolol before memory retrieval in the present study, but we cannot exclude the possible effect of propranolol on memory retrieval in the present study, and propranolol administration after retrieval should also be tested.

The present results were similar to previous studies, in which propranolol administration before reactivation decreased fear responses to the reactivated CS^13,14^. This paradigm extends previous findings, in which propranolol administration before US-induced memory reactivation generally caused a lower response to all CSs that were paired with the same US. We previously introduced a US-based memory retrieval-extinction procedure to target all diverse cues that are associated with electric shock^17^. This paradigm also effectively inhibited multiple nicotine-related memories in our recent study^19^. These studies indicate that US retrieval reactivated all memories that were associated with the US, and US-based interference may have a broader effect on the inhibition of fear memory. In addition, other studies explored the inhibitory effects of pharmacological manipulations on fear conditioning after US retrieval^16,20,23^. The US-based memory retrieval procedure with propranolol that was employed herein was effective in humans who were subjected to a single US exposure. One alternative interpretation of the present findings is that prediction errors may reactivate fear memory. Previous studies found that prediction errors were a prerequisite for memory reconsolidation in both rodents and humans^31–33^. This indicates that CS exposure renders fear memory vulnerable to the effects of propranolol, thereby reducing fear response. A low-intensity US can also induce prediction errors^34^. We speculate that US retrieval generates larger prediction errors than CS retrieval. Robust prediction errors after US retrieval may affect interference-resistant memories and interference after US retrieval could inhibit all CS-associated memory traces^35^. Altogether, these findings strongly suggest that the US-based memory retrieval interference procedure with propranolol may be more promising for fear inhibition than the CS-based procedure. Additionally, this new procedure also effectively disrupted remote fear memory. Compared with recent fear memory, remote fear memory may have a different neural basis and more significant clinical implications^36,37^.

Propranolol crosses the blood-brain barrier and acts on β-ARs in the amygdala, a brain area that is essential for emotional regulation, to interfere with the neurobiological cyclic adenosine monophosphate (cAMP)/protein kinase A (PKA)/cAMP response element binding protein (CREB) cascade that is involved in the reconsolidation of destabilized fear memories^38–40^. In the present study, we found that the US-based memory retrieval procedure with propranolol inhibited multiple CSs. One possible explanation for this finding is that the US and CS may induce differential memory reconsolidation processes. Unconditioned stimulus retrieval induced greater CREB activation in the amygdala and hippocampus than CS retrieval^20^. The endocytosis of α-amino-3-hydroxy-5-methyl-4-isoxazolepropionic acid receptors in the amygdala also plays a critical role in the inhibitory effect of US retrieval-evoked memory reconsolidation^41^. Our previous study also found that US retrieval activated distinct basolateral amygdala neuronal ensembles that encoded multiple nicotine memories^42^. Thus, US retrieval induces multiple memory traces, whereas CSs induce memory traces selectively and discretely, meaning that a major limitation of CS retrieval-induced reconsolidation is specific to the reactivated CS^16,17,43^. Another possible explanation is that hyperactivation of the amygdala and dorsal anterior cingulate cortex in response to the US occurs in human Pavlovian fear conditioning, suggesting that the amygdala and cingulate cortex are involved in processing aversive stimuli^44,45^. The present experimental design used an electric shock that was self-reported by the subjects to be “highly uncomfortable but not painful” as the appropriate US intensity to train and reactivate fear memory. Such uncomfortable stimulation is accompanied by activation of the brainstem, the thalamus, the cingulate, and sensory and insular cortices, which are involved in sensory inputs^45^. In addition, the US may induce acute stress that can interfere with memory retrieval and in turn reduce the return of fear^46^. However, the detailed mechanism of action of the US-based memory retrieval interference procedure with propranolol requires further investigation.

One important issue to consider is whether propranolol administration during memory reconsolidation changes the SCR. Studies by Soeter et al. did not observe any effects of the behavioral procedure on skin conductance discrimination^13,30,47^, but several procedural differences may explain these disparate results. First, as opposed to our neutral geometric figures, fear-relevant stimuli (e.g., spiders, guns, and other categories that can serve as a CS^+^) that capture visual attention may have rendered post-retrieval interventions ineffective on the SCR in previous studies^48^. Second, ratings of subjective distress or the presentation of a startle probe may have interfered with measurements of the SCR, which is highly sensitive to attentional processes^49^. Moreover, Soeter’s studies were limited to interventions that used a CS and not a US. Future studies need to explore whether pharmacological interventions after US presentation affect subjective distress and subjective state and trait anxiety. Third, the timing of drug administration was different. β-adrenergic receptors are critical for memory reconsolidation within a specific time window. Significant decreases in fear responses to the reactivated CS1 were observed after drug administration 1 h before reactivation but not 2 h before reactivation^14^. Thus, different concentrations of propranolol in the brain may lead to different behavioral manifestations.

The present study has several limitations. First, the generalizability of our findings may be limited because the experiments were conducted in healthy subjects. Further investigations need to test whether our procedure is effective in subclinical or clinical populations. A previous study found that disrupting the tarantula-induced memory reconsolidation by propranolol transferred avoidance behavior into approach behavior in individuals with spider phobia^23^. Another study also found that propranolol administration during reconsolidation alleviated public speaking anxiety^50^. These suggest the possibility that application of the US-based memory retrieval procedure with propranolol in anxiety and fear-related disorders may have a beneficial effect. Second, the present study used 2 weeks as a threshold to assess the sustainability of treatment efficacy and explored remote fear memory. These intervals were relatively short. Future studies need to explore longer-lasting effects of reconsolidation-focused interventions on fear memory. Third, we assessed the SCR as the outcome measure of the fear response. Other behavioral measures of US expectancy ratings and other physiological indicators (e.g., blood pressure and heart rate) should be assessed to confirm the effects of the US-based memory retrieval interference procedure on fear memory. Fourth, because of the contraindications and side effects of propranolol, it is unavoidable that the current medication-based procedure has limitations for clinical application in the future. Safer and more effective interventions still need to be explored. Fifth, in the present study, a single-blind procedure is impossible to exclude the influence of subjective bias on experimental results. A more rigorous experimental design is needed. Sixth, the neural differences between CS and US retrieval-induced reconsolidation processes are still unclear. Future studies should investigate the neural mechanisms of CS- and US-based memory retrieval interference procedures.

In conclusion, the present study introduces a new modified US-based memory retrieval interference procedure with propranolol that disrupts all fear responses and prevents the return of fear in humans. Future studies should determine the neural mechanisms that are involved in these effects and extend the procedure to clinical populations.

## Supplementary information

Supplementary Information
