# Supplementary material for: Propranolol-induced inhibition of unconditioned stimulus-reactivated fear memory prevents the return of fear in humans
Source: Transl Psychiatry. 2020 Oct 13;10:345. doi: 10.1038/s41398-020-01023-w (PMC7555531; doi:10.1038/s41398-020-01023-w)
Supplement: Supplementary file 1 — Supplementary Information [file 41398_2020_1023_MOESM1_ESM.docx]

**Supplementary Information**

**Table S1. Demographic data and shock intensity in Experiment 1.**

| **Group** |  | **No retrieval + propranolol** | **US retrieval + propranolol** | **US retrieval + placebo** | **US retrieval + 8 h + propranolol** | ***F*/*χ^2^*** | ***p*** |
| --- | --- | --- | --- | --- | --- | --- | --- |
| Gender (% female) |  | 42.86 | 53.33 | 50.00 | 57.14 | 0.62 | 0.89 |
| Age (years) |  | 23.71 ± 0.19 | 23.47 ± 0.46 | 23.88 ± 0.62 | 23.27 ± 0.44 | 0.34 | 0.80 |
| Education (years) |  | 17.71 ± 0.19 | 17.47 ± 0.46 | 17.81 ± 0.63 | 17.07 ± 0.36 | 0.55 | 0.65 |
| Height (cm) |  | 170.79 ± 1.68 | 167.20 ± 1.67 | 167.44 ± 1.63 | 168.00 ± 2.77 | 0.66 | 0.58 |
| Weight (kg) |  | 63.21 ± 2.84 | 60.50 ± 1.71 | 61.69 ± 2.39 | 60.8 ± 2.88 | 0.23 | 0.88 |
| BMI |  | 21.52 ± 0.62 | 21.64 ± 0.54 | 21.92 ± 0.63 | 21.37 ± 0.50 | 0.17 | 0.92 |
| MoCA score |  | 28.21 ± 0.28 | 28.4 ± 0.29 | 28.06 ± 0.30 | 27.8 ± 0.24 | 0.83 | 0.49 |
| Digit span test score | forward | 9.21 ± 0.21 | 9.87 ± 0.38 | 9.31 ± 0.31 | 9.53 ± 0.17 | 1.03 | 0.39 |
|  | backward | 7.50 ± 0.20 | 7.20 ± 0.53 | 7.69 ± 0.38 | 7.60 ± 0.27 | 0.33 | 0.81 |
| Shock intensity (V) |  | 59.55 ± 3.29 | 55.68 ± 3.93 | 55.51 ± 2.58 | 57.25 ± 2.98 | 0.33 | 0.80 |

The results are expressed as mean ± SEM. BMI, Body Mass Index; MoCA, Montreal Cognitive Assessment.

**Table S2. Demographic data and shock intensity in Experiment 2.**

| **Group** |  | **CS1 retrieval + propranolol** | **US retrieval + propranolol** | ***t*/*χ^2^*** | ***p*** |
| --- | --- | --- | --- | --- | --- |
| Gender (% female) |  | 58.82 | 53.33 | 0.10 | 0.76 |
| Age (years) |  | 22.94 ± 0.47 | 23.47 ± 0.46 | -0.80 | 0.43 |
| Education (years) |  | 17.71 ± 0.48 | 17.47 ± 0.46 | 0.36 | 0.72 |
| Height (cm) |  | 167.24 ± 1.81 | 167.67 ± 1.99 | -0.16 | 0.87 |
| Weight (kg) |  | 57.76 ± 2.06 | 60.93 ± 1.82 | -1.26 | 0.23 |
| BMI |  | 20.55 ± 0.41 | 21.69 ± 0.53 | -1.89 | 0.07 |
| MoCA score |  | 28.00 ± 0.34 | 28.33 ± 0.27 | -0.75 | 0.46 |
| Digit span test score | forward | 9.53 ± 0.23 | 9.87 ± 0.38 | -0.77 | 0.45 |
|  | backward | 7.76 ± 0.30 | 7.53 ± 0.50 | 0.40 | 0.68 |
| Shock intensity (V) |  | 55.69 ± 3.08 | 51.41 ± 3.28 | 0.95 | 0.35 |

The results are expressed as mean ± SEM. BMI, Body Mass Index; MoCA, Montreal Cognitive Assessment.

**Table S3. Demographic data and shock intensity in Experiment 3.**

| **Group** | |  | | **US retrieval + placebo** | | **US retrieval + propranolol** | ***t*/*χ^2^*** | ***p*** |
| --- | --- | --- | --- | --- | --- | --- | --- | --- |
| Gender (% female) | | | 52.94 | | 42.86 | | 0.42 | 0.84 |
| Age (years) | | | 23.24 ± 0.50 | | 23.67 ± 0.48 | | -0.63 | 0.54 |
| Education (years) | | | 16.18 ± 0.45 | | 16.83 ± 0.42 | | -1.07 | 0.29 |
| Height (cm) | | | 169.24 ± 1.61 | | 169.83 ± 1.85 | | -0.24 | 0.81 |
| Weight (kg) | | | 63.12 ± 2.41 | | 62.44 ± 2.76 | | 0.18 | 0.86 |
| BMI | | | 21.94 ± 0.56 | | 21.46 ± 0.55 | | 0.60 | 0.55 |
| MoCA score | | | 27.88 ± 0.31 | | 28.06 ± 0.29 | | -0.41 | 0.68 |
| Digit span test score | forward | | 8.88 ± 0.24 | | 9.17 ± 0.27 | | -0.78 | 0.44 |
|  | backward | | 7.65 ± 0.24 | | 7.72 ± 0.23 | | -0.22 | 0.82 |
| Shock intensity (V) | | | 62.25 ± 2.87 | | 62.59 ± 2.07 | | -0.10 | 0.92 |

The results are expressed as mean ± SEM. BMI, Body Mass Index; MOCA, Montreal Cognitive Assessment.
